# Assessment of Bypass of the Nearest Primary Health Care Facility Among Women in Ghana

**DOI:** 10.1001/jamanetworkopen.2020.12552

**Published:** 2020-08-12

**Authors:** Griffith Bell, Erlyn K. Macarayan, Hannah Ratcliffe, June-Ho Kim, Easmon Otupiri, Stuart Lipsitz, Lisa Hirschhorn, John Koku Awoonor-Williams, Belinda Afriyie Nimako, Anthony Ofosu, Hannah Leslie, Asaf Bitton, Dan Schwarz

**Affiliations:** 1 Ariadne Labs, Brigham and Women’s Hospital, Harvard T.H. Chan School of Public Health, Boston, Massachusetts; 2 Lancet Commission on High Quality Health Systems, Department of Global Health and Population, Harvard T.H. Chan School of Public Health, Boston, Massachusetts; 3 Division of General Internal Medicine & Primary Care, Brigham and Women’s Hospital, Boston, Massachusetts; 4 Kwame Nkrumah University of Science and Technology, Kumasi, Ghana; 5 Center for Surgery and Public Health, Brigham and Women’s Hospital, Harvard Medical School, Boston, Massachusetts; 6 Department of Medical Social Sciences, Feinberg School of Medicine, Northwestern University, Chicago, Illinois; 7 Policy Planning Monitoring and Evaluation Division, Ghana Health Service, Accra, Ghana; 8 Department of Health Care Policy, Harvard Medical School, Boston, Massachusetts

## Abstract

**Importance:**

Recent reports have highlighted that expanding access to health care is ineffective at meeting the goal of universal health coverage if the care offered does not meet a minimum level of quality. Health care facilities nearest to patient’s homes that are perceived to offer inadequate or inappropriate care are frequently bypassed in favor of more distant private or tertiary-level hospital facilities that are perceived to offer higher-quality care.

**Objective:**

To estimate the frequency with which women in Ghana bypass the nearest primary health care facility and describe patient experiences, costs, and other factors associated with this choice.

**Design, Setting, and Participants:**

This nationally representative survey study was conducted in 2017 and included 4203 households to identify women in Ghana aged 15 to 49 years (ie, reproductive age) who sought primary care within the last 6 months. Women who sought care within the past 6 months were included in the study. Data were analyzed from 2018 to 2019.

**Exposures:**

Bypass was defined as a woman’s report that she sought care at a health facility other than the nearest facility.

**Main Outcomes and Measures:**

Sociodemographic characteristics, reasons why women sought care, reasons why women bypassed their nearest facility, ratings for responsiveness of care, patient experience, and out-of-pocket costs. All numbers and percentages were survey-weighted to account for survey design.

**Results:**

A total of 4289 women met initial eligibility criteria, and 4207 women (98.1%) completed the interview. A total of 1993 women reported having sough health care in the past 6 months, and after excluding those who were ineligible and survey weighting, the total sample included 1946 women. Among these, 629 women (32.3%) reported bypassing their nearest facilities for primary care. Women who bypassed their nearest facilities, compared with women who did not, were more likely to visit a private facility (152 women [24.5%] vs 202 women [15.6%]) and borrow money to pay for their care (151 women [24.0%] vs 234 women [17.8%]). After adjusting for covariates, women who bypassed reported paying a mean of 107.2 (95% CI, 79.1-135.4) Ghanaian Cedis (US $18.50 [95% CI, $13.65-$23.36]) for their care, compared with a mean of 58.6 (95% CI, 28.1-89.2) Ghanaian Cedis (US $10.11 [95% CI, $4.85-15.35]) for women who did not bypass (*P* = .006). Women who bypassed cited clinician competence (136 women [34.3%]) and availability of supplies (93 women [23.4%]) as the most important factors in choosing a health facility.

**Conclusions and Relevance:**

The findings of this survey study suggest that bypassing the nearest health care facility was common among women in Ghana and that available services at lower levels of primary care are not meeting the needs of a large proportion of women. Among the benefits women perceived from bypassing were clinician competence and availability of supplies. These data provide insights to policy makers regarding potential gaps in service delivery and may help to guide primary health care improvement efforts.

## Key Points

**Question:**

How frequently do women in Ghana bypass the health care facility nearest their homes in favor of more distant facilities perceived to offer better care, and what factors are associated with this choice?

**Findings:**

In this survey study including 1946 reproductive-aged (ie, 16-49 years) women in Ghana in 2017, 32% of women reported bypassing their nearest health care facility. Women who bypassed sought care at hospitals and private facilities more frequently and paid nearly 2-fold in out-of-pocket costs for care compared with women who did not bypass.

**Meaning:**

These findings suggest that the services offered at many primary care facilities in Ghana may not be meeting the needs of women.

## Teaser

This survey study examines the prevalence, individual characteristics and experiences, and costs associated with bypassing the nearest health care facility among women in Ghana.

## Introduction

Recent global initiatives have embraced ambitious new goals to improve primary health care service delivery and achieve universal health coverage. Three 2018 reports^1,2,3^ have highlighted that significant work remains to be done to measure and improve quality and that merely expanding access to care is insufficient if the care provided does not meet patients’ needs.

Evidence shows that local health care facilities are frequently not used or are bypassed by individuals in favor of secondary or tertiary-level hospital facilities even when care could be managed at the local primary health care level.^4,5,6,7,8^ This underutilization of dedicated primary care facilities may lead to increased travel and cost burdens for patients, increased costs to health systems through inefficient utilization, overburdening of higher level facilities, and subversion of the goals of universal health coverage.^4,9,10,11^

The prevalence of bypassing varies widely within and between countries and health systems.^2,4,6,8,9,10,11,12,13,14,15,16,17,18,19^ Many individuals bypass what they perceive to be understaffed and poorly equipped public government-run health facilities to seek care in private facilities and hospitals.^10^ Several studies have described substantial variation in individual behavior across different health systems, particularly with regard to childbirth facilities.^4,5,6,11,14,17,20,21,22,23,24,25,26,27,28,29,30,31,32,33^

There is limited evidence characterizing the extent of primary health care bypass in Ghana and West Africa in general. A better understanding of these bypass behaviors may provide insights into local primary health care improvement efforts for policy makers and facility managers. Recent surveys by Performance Monitoring and Accountability 2020 (PMA2020)^34,35^ provide nationally-representative data on care seeking and utilization of primary health care in Ghana. Using these data, we explored several questions. First, what is the prevalence of bypassing primary health care facilities in Ghana? Second, how did bypassing primary health care facilities vary by individual demographic and geographic characteristics? Third, what factors were associated with why and where individuals choose to seek care? Fourth, did the self-reported experience of individuals who bypassed differ from those who did not bypass? Finally, how were individuals’ financial costs associated with bypassing?

## Methods

This study was approved by School of Medical Sciences, Komfo Anokye Teaching Hospital Committee on Human Research Publications and Ethics, the Partners Human Research Committee, and the Johns Hopkins School of Public Health institutional review board. All study participants provided written informed consent. This study is reported following the American Association for Public Opinion Research (AAPOR) reporting guideline.

### Study Area and Data Collection

Extended description of the data collection methods used by PMA2020 can be found elsewhere.^34,35^ The survey was designed to provide nationally representative data on key health and development indicators in Ghana. The survey was powered to estimate the modern contraceptive prevalence in women of reproductive age (ie, 15-49 years). Data were collected in partnership with the Kwame Nkrumah University of Science and Technology and included a specially designed primary health care module integrated in the existing PMA2020 facility and household surveys used to track progress toward family planning use targets. Surveys were administered in English and local languages. To obtain the data, the Ghana Statistical Service selected 100 enumeration areas across all 10 regions with probability proportional to size using a master sampling frame stratified by urban-rural areas. Within these enumeration areas, 42 households were selected using a random start method to complete the household survey, and all members of the household were surveyed. All data used in this study were collected during round 6 of the PMA2020 survey conducted in late 2017.

### Bypass Definition

In this study, bypass was defined as a woman’s report that she sought care at a health facility other than the nearest facility to her residence (“Is this facility the closest facility to your place of residence?” with binary yes or no answer). The woman was then categorized as having bypassed her nearest facility or not.

### Variables

We examined 5 categories of variables and their association with bypassing: demographic characteristics (ie, age, marital status, educational attainment, household wealth, residence [urban or rural]), utilization of care (ie, facility type visited, reasons for seeking care, for whom the care was sought, factors considered most important in the choice of health facility), financial costs, responsiveness of care,^36^ and self-reported patient experience. A responsiveness index was calculated as the mean score from a woman’s rating of all responsiveness questions. The responsiveness index was divided into quintiles and dichotomized into highest quintile vs all others. A detailed list of variables and descriptions for each category can be found in the eAppendix in the Supplement.

### Statistical Analysis

All women who responded to the survey and had visited a health facility in the last 6 months were included in the analyses. To generate nationally representative, population-based estimates, we used survey-weighted summary statistics that accounted for the multistage clustered survey design in all analyses.

We used Poisson regression models with a log-link and robust SEs to estimate the associations of bypass (main variable) with women’s self-reported experience of their care. Outcomes were dichotomized as highest or most positive ratings vs all other ratings combined (a top-box categorization). The exponentiated regression coefficients from the Poisson regression can be interpreted as relative risks (RRs) comparing the proportion of those giving the highest rating between women who bypassed and those who did not. We used linear regression with robust SEs to estimate mean out-of-pocket costs paid by women who bypassed and those who did not. All models were first fit unadjusted for other variables, and then with adjustment for demographic and geographic factors and reasons for seeking care (eAppendix in the Supplement). *P* values were 2-sided and reserved for model-based analyses and not descriptive statistics to avoid issues of multiple testing. Statistical significance was set at *P* < .05. We performed analyses in Stata version 15.1 (StataCorp) and SAS version 9.1 (SAS Institute) statistical software. Data were analyzed from 2018 to 2019.

## Results

A total of 4289 women met eligibility criteria, and 4207 women (98.1%) were available, consented to the interview, and completed the interview. All 4207 women were asked whether they had visited a health care facility within 6 months prior to the survey. Of those who answered the health care facility question, 1993 women (46.7%) answered yes, and thus completed the subsequent primary health care–specific survey questions. All women answered the bypass question. Of women who had sought health care in the past 6 months, 28 (1.4%) were excluded because the sampled household was not their primary residence. Therefore, the sample of interest for this study included 1965 women who had sought care in the last 6 months and lived in the sample household, which was 1946 women after reweighting to account for the survey design (eFigure in the Supplement). Table 1 provides summary statistics for the sample, stratified by bypass status. Using methods accounting for the multistage sampling design and rounded to the nearest integer, 629 women (32.3%) reported bypassing their nearest facility to seek care.

**Table 1.** Health Facility Utilization Among Women in Ghana

Characteristic	Respondents, No. (%)^a^		
	Did not bypass nearest facility (n = 1317)	Bypassed nearest facility (n = 629)	Total (N = 1946)
Age, y			
15-24	398 (30.2)	152 (24.2)	550 (28.3)
25-34	501 (38.1)	236 (37.6)	738 (37.9)
35-49	418 (31.7)	240 (38.2)	658 (33.8)
Wealth quintile			
Lowest	289 (21.9)	114 (18.1)	403 (20.7)
Lower	259 (19.7)	112 (17.9)	371 (19.1)
Middle	259 (19.6)	132 (20.9)	390 (20.0)
Higher	291 (22.1)	133 (21.2)	424 (21.8)
Highest	220 (16.7)	138 (21.9)	358 (18.4)
Education			
Never attended	220 (16.7)	106 (16.8)	326 (16.7)
Primary	233 (17.7)	119 (18.9)	352 (18.1)
Middle junior secondary school	521 (39.6)	232 (36.9)	754 (38.7)
Secondary junior secondary school	237 (18.0)	109 (17.3)	345 (17.8)
Higher	106 (8.1)	63 (10.1)	170 (8.7)
Marital status			
Currently married	700 (53.2)	339 (53.8)	1039 (53.4)
Currently living with partner	211 (16.0)	104 (16.5)	315 (16.2)
Divorced	101 (7.7)	48 (7.6)	149 (7.6)
Widow	35 (2.6)	16 (2.6)	51 (2.6)
Never married	270 (20.5)	122 (19.4)	393 (20.2)
Residence			
Urban	648 (49.2)	324 (51.4)	971 (49.9)
Rural	670 (50.8)	305 (48.6)	975 (50.1)
Region			
Ashanti	256 (19.4)	144 (22.9)	400 (20.6)
Brong Ahafo	75 (5.7)	36 (5.7)	110 (5.7)
Central	98 (7.4)	80 (12.7)	178 (9.1)
Eastern	184 (14.0)	45 (7.2)	229 (11.8)
Greater Accra	210 (15.9)	81 (12.9)	291 (14.9)
Northern	126 (9.6)	76 (12.1)	202 (10.4)
Upper East	97 (7.4)	36 (5.7)	133 (6.8)
Upper West	60 (4.5)	12 (2.0)	72 (3.7)
Volta	78 (5.9)	45 (7.1)	123 (6.3)
Western	134 (10.2)	74 (11.7)	207 (10.7)
Facility type visited			
Community-based health planning and services	196 (15.2)	14 (2.3)	210 (11.0)
Government hospital or polyclinic	451 (34.9)	326 (52.6)	777 (40.6)
Government health center	366 (28.3)	97 (15.7)	464 (24.2)
Private hospital or clinic	202 (15.6)	152 (24.5)	353 (18.5)
Other	78 (6.0)	31 (4.9)	108 (5.7)
Owns a bicycle	358 (27.2)	143 (22.8)	501 (25.8)
Owns a car	115 (8.8)	66 (10.6)	182 (9.3)
Ability to pay for visit			
Very easy	335 (25.6)	166 (26.4)	500 (25.8)
Easy	628 (48.0)	235 (37.4)	863 (44.6)
Difficult	231 (17.6)	143 (22.7)	373 (19.3)
Very difficult	115 (8.8)	85 (13.5)	200 (10.3)
Services covered by insurance	994 (75.5)	468 (74.4)	1462 (75.1)
Had to borrow money to pay for visit	234 (17.8)	151 (24.0)	385 (19.8)
Paid any fees for family planning last 12 mo	316 (62.7)	112 (50.3)	428 (58.9)
Confidence that if you became very sick tomorrow, you would be able to receive effective treatment from the health system			
Not at all confident	42 (3.2)	14 (2.2)	56 (2.9)
Not very confident	96 (7.3)	39 (6.1)	135 (7.0)
Somewhat confident	260 (19.8)	112 (17.8)	372 (19.2)
Very confident	915 (69.7)	463 (73.8)	1378 (71.0)
Rating of the amount of time spent with clinician			
Poor	18 (1.4)	6 (0.9)	23 (1.2)
Fair	93 (7.1)	45 (7.1)	138 (7.1)
Good	619 (47.0)	238 (37.9)	857 (44.0)
Very good	397 (30.1)	211 (33.5)	608 (312)
Excellent	190 (14.4)	130 (20.6)	320 (16.4)
Treated by the same clinician each time			
Never	92 (7.0)	71 (11.3)	163 (8.4)
Rarely	507 (38.6)	259 (41.2)	766 (39.4)
Frequently	382 (29.1)	193 (30.6)	575 (29.6)
Always	332 (25.3)	106 (16.9)	438 (22.6)
Sought care for self	882 (67.0)	465 (74.0)	1348 (69.2)
Sought care for child	573 (43.5)	231 (36.7)	804 (41.3)
Sought care for other	72 (5.5)	34 (5.4)	106 (5.4)

^a^ All data are survey weighted. Counts may not sum to the denominator of the column due to the rounding of survey-weighted data.

### Demographic Characteristics Among Women Who Bypassed

A total of 305 of 975 rural women (31.3%) bypassed their nearest health facility, compared to 324 of 971 urban women (33.3%) (Table 1). Women in urban areas tended to be wealthier than those in rural areas, with 335 women (34.5%) in the highest wealth quintile compared with 23 women (2.3%) in rural areas (eTable 1 in the Supplement). There were few women in the highest quintile of wealth in rural areas; however, these women had higher levels of bypassing than the wealthiest women in urban areas (eTable 1 in the Supplement).

Women who bypassed their nearest facility were more likely to be seeking care for themselves (as opposed to for a child or other family member or friend) than women who did not bypass (465 women [73.9%] vs 882 women [67.0%]) (Table 2). Women who went to their nearest facility more frequently reported going for vaccinations than women who bypassed (129 women [10.0%] vs 28 women [4.8%]); however, overall reasons for care-seeking were generally similar between women who bypassed and those who did not (Table 2), suggesting that differing clinical needs were not the major driver of bypass. In particular, the proportions of women who sought care owing to a community health worker referral were similar among women who did not bypass and those who did (13 women [1.0%] vs 4 women [0.7%]). Some women may have self-referred to a more distant facility for strategic reasons. To further examine these patterns, we performed a sensitivity analysis in which we examined strategic bypass. This analysis included only women who bypassed their nearest facility because it was closed or did not offer their desired services. After looking only at this selected group of women, we found no significant differences in our primary findings (eTable 2 in the Supplement).

**Table 2.** Reasons Women Reported for Seeking Care

Reasons care was sought	Respondents, No./total (%)^a^		
	Did not bypass nearest facility	Bypassed nearest facility	Total
Family planning			
No	1181/1292 (91.4)	557/587 (95.0)	1739/1879 (92.5)
Yes	111/1292 (8.6)	30/587 (5.0)	140/1879 (7.5)
Maternal needs			
No	1034/1292 (80.0)	473/587 (80.6)	1507/1879 (80.2)
Yes	258/1292 (20.0)	114/587 (19.4)	372/1879 (19.8)
Vaccine			
No	1163/1292 (90.0)	559/587 (95.2)	1722/1879 (91.6)
Yes	129/1292 (10.0)	28/587 (4.8)	157/1879 (8.4)
Fever			
No	730/1292 (56.5)	356/587 (60.6)	1086/1879 (57.8)
Yes	562/1292 (43.5)	231/587 (39.4)	793/1879 (42.2)
Sickness			
No	1015/1292 (78.5)	447/587 (76.2)	1462/1879 (77.8)
Yes	277/1292 (21.5)	140/587 (23.8)	417/1879 (22.2)
Referred by community health worker			
No	1279/1292 (99.0)	583/587 (99.3)	1862/1879 (99.1)
Yes	13/1292 (1.0)	4/587 (0.7)	17/1879 (0.9)
Snakebite			
No	1289/1292 (99.8)	586/587 (99.8)	1875/1879 (99.8)
Yes	3/1292 (0.2)	1/587 (0.2)	4/1879 (0.2)
Injury			
No	1257/1292 (97.3)	570/587 (97.1)	1828/1879 (97.3)
Yes	34/1292 (2.7)	17/587 (2.9)	51/1879 (2.7)
Blood pressure			
No	1227/1292 (95)	548/587 (93.4)	1775/1879 (94.5)
Yes	65/1292 (5.0)	39/587 (6.6)	104/1879 (5.5)
Diabetes			
No	1283/1292 (99.3)	580/587 (98.8)	1863/1879 (99.1)
Yes	9/1292 (0.7)	7/587 (1.2)	16/1879 (0.9)
HIV			
No	1288/1292 (99.7)	583/587 (99.4)	1872/1879 (99.6)
Yes	4/1292 (0.3)	4/587 (0.6)	7/1879 (0.4)
Eye issue			
No	1275/1292 (98.7)	573/587 (97.6)	1848/1879 (98.3)
Yes	17/1292 (1.3)	14/587 (2.4)	31/1879 (1.7)
Respiratory condition			
No	1243/1292 (96.2)	565/587 (96.2)	1807/1879 (96.2)
Yes	49/1292 (3.8)	22/587 (3.8)	72/1879 (3.8)
Abdominal issue			
No	1230/1292 (95.2)	546/587 (92.9)	1776/1879 (94.5)
Yes	62/1292 (4.8)	41/587 (7.1)	103/1879 (5.5)
Concern about new symptom			
No	1232/1292 (95.3)	546/587 (93)	1777/1879 (94.6)
Yes	60/1292 (4.7)	41/587 (7.0)	102/1879 (5.4)

^a^ All data are survey weighted. Counts may not sum to the denominator of the column due to the rounding of survey-weighted data.

Rural women who bypassed typically did not seek care at community-based health planning and services facilities (eTable 1 in the Supplement). Instead, women who bypassed frequently sought care at hospitals or polyclinics and private facilities. Wealthy rural women were also much more likely to bypass their nearest facility than their urban counterparts (eTable 1 in the Supplement).

### Factors Associated With Where Women Sought Care

Regardless of bypass status, the 3 highest-ranking factors in choice of health care were competency of the clinician (with *clinician* defined as anyone who provides clinical care) (346 women [27.4%]), well-supplied facility (273 women [21.7%]), and respect from the clinician (156 women [12.4%]) (Table 3). Among women who bypassed, 136 women (34.3%) reported competence of the clinician at that facility as being the most important factor influencing their decision, compared with 210 women (24.3%) who did not bypass. The distance of the facility from the respondent’s home was another key difference between those who bypassed and those who did not, with just 8 women (2.1%) who bypassed listing it as the most important factor in choosing a care facility, compared with 91 women (10.5%) who did not bypass. Women who bypassed reported a variety of reasons why they bypassed their closest facility, of which the most commonly reported reason was that their closest facility did not provide the services that they needed (234 of 479 respondents [48.9%]) (eTable 2 in the Supplement).

**Table 3.** Most Important Factors Reported in Choice of Health Facility

Factor	Respondents, No. (%)^a^		
	Did not bypass nearest facility (n = 1309)	Bypassed nearest facility (n = 621)	Total (N = 1930)
**Most important overall**			
Competency of clinician	210 (24.3)	136 (34.3)	346 (27.4)
Facility well supplied	181 (20.9)	93 (23.4)	273 (21.7)
Respect from clinician	109 (12.6)	47 (11.9)	156 (12.4)
Cost of treatment	104 (12.0)	34 (8.6)	138 (10.9)
Waiting time	71 (8.2)	30 (7.5)	101 (8.0)
Privacy	44 (5.1)	23 (5.9)	68 (5.4)
Cleanliness of facility	23 (2.7)	12 (3.1)	36 (2.8)
Distance	91 (10.5)	8 (2.1)	100 (7.9)
Choice of clinician	5 (0.6)	7 (1.6)	11 (0.9)
Knowledge of clinician	6 (0.7)	4 (0.9)	10 (0.8)
Other	1 (0.1)	2 (0.4)	3 (0.2)
Cost of visit	17 (2.0)	1 (0.3)	18 (1.5)
Traditional medicine practiced	2 (0.2)	0	2 (0.1)
Total	867 (100)	396 (100)	1263 (100)
**Described as important for choosing facility**			
Wait time			
No	946 (72.3)	470 (75.6)	1415 (73.3)
Yes	363 (27.7)	152 (24.4)	515 (26.7)
Distance			
No	939 (71.8)	561 (90.4)	1501 (77.8)
Yes	369 (28.2)	60 (9.6)	429 (22.2)
Cleanliness			
No	931 (71.1)	456 (73.3)	1386 (71.8)
Yes	378 (28.9)	166 (26.7)	544 (28.2)
Respect from clinician			
No	840 (64.2)	394 (63.4)	1234 (63.9)
Yes	469 (35.8)	228 (36.6)	696 (36.1)
Competence of clinician			
No	776 (59.3)	269 (43.3)	1045 (54.2)
Yes	532 (40.7)	352 (56.7)	885 (45.8)
Privacy			
No	1103 (84.3)	527 (84.9)	1631 (84.5)
Yes	206 (15.7)	94 (15.1)	299 (15.5)
Well-supplied facility			
No	872 (66.6)	392 (63.0)	1263 (65.5)
Yes	437 (33.4)	230 (37.0)	667 (34.5)
Cost of treatment			
No	925 (70.7)	461 (74.1)	1386 (71.8)
Yes	383 (29.3)	161 (25.9)	544 (28.2)
Cost of visit			
No	1223 (93.5)	607 (97.6)	1830 (94.8)
Yes	86 (6.5)	15 (2.4)	100 (5.2)
Choice of clinician			
No	1269 (96.9)	593 (95.5)	1862 (96.5)
Yes	40 (3.1)	28 (4.5)	68 (3.5)
Knowledge of clinician			
No	1277 (97.5)	607 (97.7)	1884 (97.6)
Yes	32 (2.5)	14 (2.3)	46 (2.4)
Traditional medicine practiced			
No	1305 (99.7)	618 (99.4)	1922 (99.6)
Yes	4 (0.3)	4 (0.6)	8 (0.4)

^a^ All data are survey weighted. Counts may not sum to the denominator of the column due to the rounding of survey-weighted data.

### Self-reported Experience of Women Who Bypassed vs Those Who Did Not

Women who bypassed generally reported receiving more responsive care (Table 4). In adjusted Poisson regression models, women who bypassed were more likely to rate the following characteristics as excellent compared with nonexcellent ratings: clinician choice (RR, 1.47 [95% CI, 1.13-1.90]; *P* = .004) and cleanliness (RR, 1.36 [95% CI, 1.08-1.71]; *P* = .01). After covariate adjustment, women who bypassed were less likely to report excellent physical health (RR, 0.87 [95% CI, 0.76-1.00]; *P* = .04). The full list of adjustment variables is presented in Table 4.

**Table 4.** Highest Ratings of Responsiveness, PROMs, and Affordability of Care Comparing Women Who Bypassed Their Nearest Health Care Facility With Those Who Did Not Using Poisson Regression With a Log-Link and Robust SEs

Rating	Unadjusted model		Adjusted model^a^	
	Relative risk (95% CI)	*P* value	Relative risk (95% CI)	*P* value
Responsiveness				
Wait rating	1.08 (0.72-1.62)	.71	0.99 (0.67-1.47)	.98
Respect from clinician	1.41 (0.97-2.06)	.07	1.14 (0.83-1.55)	.41
Involvement	1.70 (1.03-2.79)	.04	1.23 (0.77-1.98)	.38
Provider choice	1.64 (1.08-2.50)	.02	1.47 (1.13-1.90)	.004
Cleanliness	1.76 (1.26-2.47)	.001	1.36 (1.08-1.71)	.01
Understand clinician	1.61 (1.06-2.43)	.02	1.24 (0.86-1.77)	.25
Privacy	1.73 (1.12-2.68)	.01	1.37 (1.00-1.87)	.05
Responsiveness index	1.56 (1.03-2.36)	.04	1.25 (0.92-1.70)	.14
PROM				
Excellent quality of care	1.21 (1.04-1.41)	.01	1.04 (0.94-1.17)	.44
Overall excellent quality of care received	1.14 (0.98-1.34)	.10	0.99 (0.88-1.10)	.80
Physical health rating	0.95 (0.80-1.14)	.57	0.87 (0.76-1.00)	.04
Mental health rating	1.06 (0.83-1.34)	.64	0.93 (0.76-1.13)	.45
Recommend this facility to others	1.01 (0.99-1.03)	.37	1.01 (0.99-1.03)	.38
Affordability of care				
Had to borrow money or sell things to pay for visit	1.35 (1.04-1.74)	.02	1.36 (1.10-1.68)	.005

Abbreviation: PROM, patient-reported outcome measure.

^a^ Adjusted for age, wealth quintile, highest level of education attained, urban or rural neighborhood, confidence that if you became very sick tomorrow that you would be able to receive effective treatment from the health system, taken care of by the same clinician each visit, reason for seeking care, person for whom care was sought, facility type, and region.

### Costs Associated With Bypassing

Women who bypassed were more likely to have borrowed money to pay for their care (RR, 1.36 [95% CI, 1.10-1.68]; *P* = .005) (Table 4). Using linear regression and adjusting for reasons for seeking care and other factors, women who bypassed reported paying a mean of 107.2 (95% CI, 79.1-135.4) Ghanaian Cedis (US $18.50 [95% CI, $13.65-$23.36]) for their care, compared with a mean of 58.6 (95% CI, 28.1-89.2) Ghanaian Cedis (US $10.11 [95% CI, $4.85-15.35]) for women who did not bypass (*P* = .006) (Table 5). Even after adjustment for other factors, including reason for seeking care, women who bypassed paid nearly 2-fold as much for their care as those who did not.

**Table 5.** Mean Costs of Care Using Generalized Linear Models With Robust SEs

Bypassed nearest facility	Unadjusted model		Adjusted model^a^	
	Cost, GH₵ (95% CI)	*P* value	Cost, GH₵ (95% CI)	*P* value
No	53.1 (29.3-76.3)	<.001	58.6 (28.1-89.2)	.006
Yes	117.0 (74.1-159.9)		107.2 (79.1-135.4)	

Abbreviation: GH₵, Ghanaian Cedi.

^a^ Adjusted for age, wealth quintile, highest level of education attained, urban or rural neighborhood, confidence that if you became very sick tomorrow, you would be able to receive effective treatment from the health system, cared for by the same clinician each visit, reason for seeking care, person for whom care was sought, facility type, and region.

## Discussion

This survey study used a nationally representative survey to estimate the countrywide prevalence of health facility bypass in Ghana. Unlike previous studies that focused on childbirth or emergency services, our analysis focused on a general population’s experience of primary care and offers insights into who bypasses, why, and the outcomes associated with their care. We found that bypassing was relatively common: 32.3% of women reported bypassing. Moreover, bypassing was mostly owing to the knowledge that the desired services were not available at the closest facility. Women who bypassed cited clinician competence as the most important factor in choosing a facility more frequently than women who did not bypass, indicating that women highly value competent clinicians and are willing to travel farther to get them. In rural areas, wealthier women tended to bypass more frequently, suggesting differences in behavior and functional access between socioeconomic strata.

Women who bypassed paid more out of pocket for their care than women who did not bypass. To our knowledge, this is the first study to examine costs associated with bypass in this context. Other studies have examined discrete choice experiments of health facility utilization and quality and found that patients are generally willing to pay more for higher-quality services, although how much more is greatly dependent on context.^20,21^ Kahabuka et al^7^ found that caretakers in Tanzania frequently bypassed nearest facilities for their children owing to lack of diagnostic equipment, insufficient drug stocks, limited hours, and poor services—similar to our findings. However, comparisons of bypass across countries can vary widely depending on the service being sought and the distribution of facilities offering that service. For example, in a 2020 study of family planning in Tanzania,^37^ 67% of women bypassed their nearest facility to seek contraception. The nearest facility was a mean of 1.2 km away, while the chosen facility was a mean of 2.9 km away. In contrast, a 2016 study of utilization of childbirth facilities in Ghana^38^ found that women lived a median 3.3 km from their nearest birth facility, with the nearest hospital 13.9 km away (bypass was not assessed). These differences reflect heterogeneities in opportunity for choice of facility, proximity of facilities offering a desired service, and burden associated with bypassing the nearest facility. Our population of women was also fairly young and as such, less likely to be seeking care for the types of chronic conditions and serious illnesses that might be associated with referral to hospitals.

Women generally reported similar reasons for seeking care whether they bypassed their nearest facility or not. This may suggest that many women’s reasons for bypassing were less associated with the nature of their underlying health concerns and more associated with their perception of quality of care or experiences at the facility. There are limited published data from other low- and middle-income countries on differences between individuals who bypass and those who do not in reasons why individuals might seek care. A 2011 study by Gauthier et al^11^ examined reasons for seeking care by facility type in Chad and reported that malaria and diarrhea were the primary reasons for seeking care; however, the study did not assess whether these reasons differed by bypass status.

Previous studies^4,6,9,10,11,18,19,39^ have suggested that bypass is frequently driven by perceptions of the quality of care offered. In our study, women who bypassed were more likely to rate their clinician choice, cleanliness of the facility, and privacy as excellent. However, despite rating these aspects of their facility highly, women who bypassed were no more likely to report excellent ratings for the overall quality of their care or to recommend the facility highly to others. This finding may be associated with differential expectations of what is considered to be acceptable care, with individuals who bypass having higher expectations than whose who do not.^40^ In previous studies,^18,41,42^ vignettes describing aspects of care indicated that individuals have wide variation in what they consider to be acceptable care, particularly those who are poorer and less educated.

Taken in the context of a growing emphasis on quality in health care delivery, our results have important policy and public health implications. They suggest that bypassing is common, expensive, and frequently associated with perceptions that the closest facilities do not provide the desired services. Women frequently reported that the competence of their clinician was of highest importance, and were most likely to bypass community-based health planning and services facilities, which do not have physicians on staff. Increased staff training, higher quality of care, and range and readiness of available services may decrease bypassing, reducing costs to patients.^7^ Further research is necessary to identify which specific services can be brought closer to patients, if possible. Our results also indicate an opportunity to improve the system’s efficiency in being a point of first contact for patients, per Starfield’s four pillars of primary care^43^ (ie, first contact, continuous care, comprehensive care, and coordinated care). While further research in this area is essential, improving the quality of care at local levels may help toward building more equitable, higher quality primary health care systems that better address patients’ needs.^44,45^

### Limitations

Our study has limitations. First, this is a cross-sectional survey designed to be nationally representative of reproductive-aged women in Ghana, so our findings should be interpreted with caution and may not be generalizable to other subpopulations. Second, our definition of bypass was based on women’s self-report of not visiting their nearest facility, which may be inaccurate, as many women may have been unaware of precise distances between their home and facilities. However, this definition has been used in previous studies, and our assumption is that women’s perception of distance, rather than actual distance, is likely to be more relevant to their choice of health care facility.^6,9,19,30^ Third, although our study focused on women who visited a facility, some women reported that they were unable to visit a health facility altogether, despite a desire for care. Fourth, because our study collected only limited data on reasons why women may have bypassed their closest facility, care should be taken in drawing definitive policy and public health conclusions based on our findings alone.

## Conclusions

In this survey study of health care utilization among reproductive-aged women in Ghana, we found that bypass was relatively common in Ghana, with nearly one-third of women bypassing the facility closest to their home. Women who bypassed their nearest health facility cited the competence of the clinician most frequently as the most important factor in facility choice. Women who bypassed were also more likely to visit hospitals and private clinics than women who did not bypass. Women who bypassed paid nearly 2-fold as much for their care as women who did not bypass. These data provide insights that may be helpful for policy makers and facility managers in Ghana as they strive to achieve high-quality universal health care for the population.

## Notes

Supplement. **eAppendix.** Detailed Description of Variables **eFigure.** Extent of Bypassing of Health Care Facilities in Ghana in 2017 **eTable 1.** Ghanaian Health Facility Utilization by Urban and Rural Neighborhood **eTable 2.** Reasons Given for Why Women Bypassed Their Nearest Facility
