# Supplementary material for: Assessment of Bypass of the Nearest Primary Health Care Facility Among Women in Ghana
Source: JAMA Netw Open. 2020 Aug 12;3(8):e2012552. doi: 10.1001/jamanetworkopen.2020.12552 (PMC7424402; doi:10.1001/jamanetworkopen.2020.12552)
Supplement: Supplement. — eAppendix. Detailed Description of Variables eFigure. Extent of Bypassing of Health Care Facilities in Ghana in 2017 eTable 1. Ghanaian Health Facility Utilization by Urban and Rural Neighborhood eTable 2. Reasons Given for Why Women Bypassed Their Nearest Facility [file jamanetwopen-3-e2012552-s001.pdf]

Supplementary Online Content

Bell G, Macarayan EK, Ratcliffe H, et al. Assessment of bypass of the nearest primary health care facility among women in Ghana. *JAMA Netw Open*. 2020;3(8):e2012552. doi:10.1001/jamanetworkopen.2020.12552

eAppendix. Detailed Description of Variables
eFigure. Extent of Bypassing of Health Care Facilities in Ghana in 2017
eTable 1. Ghanaian Health Facility Utilization by Urban and Rural Neighborhood
eTable 2. Reasons Given for Why Women Bypassed Their Nearest Facility

This supplementary material has been provided by the authors to give readers additional information about their work.

© 2020 Bell G et al. *JAMA Network Open*

eAppendix. Detailed Description of Variables

Demographic variables:

1. Age
2. Highest level of educational attainment
3. Marital status
4. Region of residence
5. Residence (urban or rural)
6. Wealth index (calculated from a list of household possessions and dwelling characteristics, and divided into quintiles)

Utilization:

1. The facility type visited (government hospital or polyclinic, private hospital or health clinic, community-based health planning and services (CHPS) compound, government health center, or other—for example, family planning clinic or maternity home)
2. The reason for seeking care, classified as acute, chronic, or preventive
3. Whether the care sought was for the respondent, their children, or others
4. Patient's reasons for seeking care
5. Factors patients felt were most important in their choice of health facility

Financial:

1. Whether patients borrowed money or sold things to afford the cost of the visit,
2. The average amount patients paid out of pocket,
3. Whether patients found the amount paid for the visit was financially difficult for them,
4. Whether patients had any type of health insurance,

Responsiveness of care (patient's rating of 7 domains of provider responsiveness, according to WHO criteria): (31)

1. Dignity *(How would you rate the level of respect the provider showed you?)*
2. Autonomy *(How would you rate your experience of being involved in making decisions for your treatment?)*
3. Choice of provider *(How would you rate the ease with which you could see a health care provider you were happy with?)*
4. Confidentiality *(How would you rate the way that health services ensured that you could talk privately to providers?)*
5. Quality of basic amenities/ surroundings/ environment *(How would you rate the cleanliness of the facility?)*
6. Communication *(How would you rate the provider's availability to explain things in a way that you could understand?)*
7. Prompt attention *(How would you rate the length of wait time at the facility before you were seen?)*
8. Responsiveness index (a weighted average of scores for all responsiveness questions)

Self-reported experience:

1. Patient satisfaction
    - Recommendation of the facility to others (very unlikely, somewhat unlikely, somewhat likely, very likely to recommend)
    - Overall rating of care received at the facility (poor, fair, good, very good, excellent)
    - How well care at the facility met the respondent's health needs (poor, fair, good, very good, excellent),
2. Patient-reported outcomes
    - Overall self-rated health (poor, fair, good, very good, excellent), as assessed by the question *"Overall, would you say your health is…"*
    - Self-rated mental health (poor, fair, good, very good, excellent), as assessed by the question *"Overall, how would you rate your mental health, including your mood and your ability to think?"*

© 2020 Bell G et al. *JAMA Network Open*

eFigure. Extent of Bypassing of Health Care Facilities in Ghana in 2017

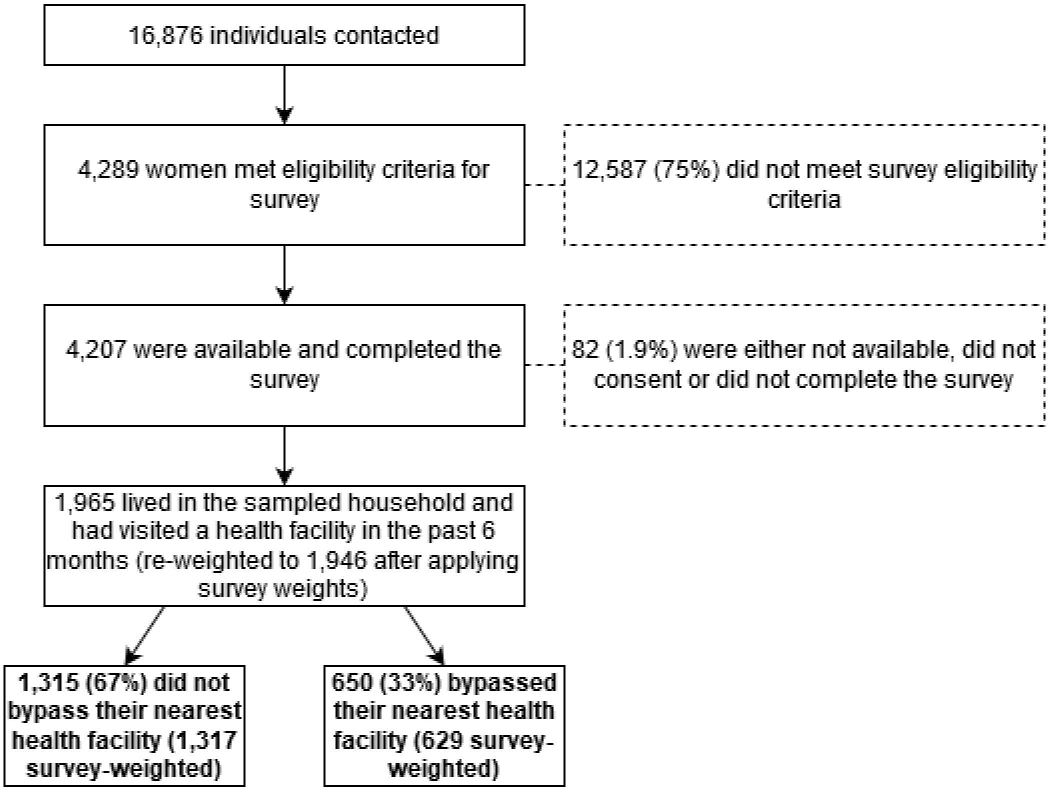

© 2020 Bell G et al. *JAMA Network Open*

eTable 1. Ghanaian Health Facility Utilization by Urban and Rural Neighborhood

		Women who visited a health facility (N=1,946)													
Characteristics		Overall n=1,946						Rural (N=975)				Urban (n=971)			
		Total		Total Rural		Total Urban		Did not bypass		Bypassed nearest facility		Did not bypass		Bypassed nearest facility	
		N	Column %	N	Column %	N	Column %	N	Row %	N	Row %	N	Row %	N	Row %
Total		1,946	100%	975	100%	971	100%	669	68.7%	305	31.3%	648	66.7%	324	33.3%
Age category															
	15-24	550	28.3%	316	32.4%	234	24.1%	234	74.1%	82	25.9%	164	70.1%	70	29.9%
	25-34	738	37.9%	330	33.8%	408	42.0%	232	70.5%	97	29.5%	269	65.9%	139	34.1%
	35-44	493	25.3%	240	24.6%	253	26.1%	155	64.9%	84	35.1%	168	66.4%	85	33.6%
	45+	165	8.5%	89	9.1%	76	7.8%	44	51.8%	41	48.2%	46	60.5%	30	39.5%
Wealth quintile															
	Lowest quintile	403	20.7%	373	38.2%	30	3.1%	263	70.6%	110	29.4%	26	85.9%	4	14.1%
	Lower quintile	371	19.1%	291	29.8%	80	8.3%	205	70.4%	86	29.6%	54	67.4%	26	32.6%
	Middle quintile	390	20.0%	204	20.9%	186	19.2%	146	71.4%	58	28.6%	113	60.6%	73	39.4%
	Higher quintile	424	21.8%	84	8.6%	340	35.0%	48	56.8%	36	43.2%	243	71.5%	97	28.5%
	Highest quintile	358	18.4%	23	2.4%	335	34.5%	8	34.7%	15	65.3%	212	63.3%	123	36.7%
Education (highest level attended)															
	Never attended	326	16.7%	234	24.0%	92	9.4%	160	68.6%	73	31.4%	60	64.9%	32	35.1%
	Primary	352	18.1%	234	24.0%	118	12.2%	160	68.4%	74	31.6%	73	61.8%	45	38.2%
	Middle/JSS	754	38.7%	379	38.8%	375	38.6%	265	70.0%	114	30.0%	257	68.4%	119	31.6%
	Secondary/JSS	345	17.8%	112	11.5%	234	24.1%	75	67.4%	36	32.6%	161	69.0%	72	31.0%
	Higher	170	8.7%	17	1.8%	153	15.7%	9	53.5%	8	46.5%	97	63.8%	55	36.2%
Marital status															
	Currently living with partner	315	16.2%	168	17.3%	146	15.1%	119	70.7%	49	29.3%	92	62.7%	55	37.3%
	Currently married	1039	53.4%	554	56.8%	485	50.0%	373	67.3%	181	32.7%	328	67.6%	157	32.4%

© 2020 Bell G et al. *JAMA Network Open*

	Divorced	149	7.6%	66	6.8%	83	8.5%	45	68.5%	21	31.5%	56	67.3%	27	32.7%
	Never married	393	20.2%	152	15.6%	240	24.7%	111	72.5%	42	27.5%	160	66.6%	80	33.4%
	Widow	51	2.6%	34	3.5%	17	1.7%	22	64.7%	12	35.3%	13	74.2%	4	25.8%
Region															
	Ashanti	400	20.6%	141	14.4%	259	26.7%	98	69.6%	43	30.4%	158	61.0%	101	39.0%
	Brong Ahafo	110	5.7%	45	4.6%	65	6.7%	35	76.5%	11	23.5%	40	61.4%	25	38.6%
	Central	178	9.1%	114	11.7%	63	6.5%	68	59.4%	46	40.6%	30	47.1%	33	52.9%
	Eastern	229	11.8%	107	11.0%	122	12.6%	71	66.6%	36	33.4%	113	92.2%	10	7.8%
	Greater Accra	291	14.9%	38	3.9%	252	26.0%	32	82.4%	7	17.6%	178	70.5%	74	29.5%
	Northern	202	10.4%	112	11.5%	90	9.3%	66	59.3%	46	40.7%	60	66.5%	30	33.5%
	Upper East	133	6.8%	118	12.1%	16	1.6%	88	75.3%	29	24.7%	9	55.6%	7	44.4%
	Upper West	72	3.7%	60	6.2%	12	1.3%	49	82.2%	11	17.8%	10	85.7%	2	14.3%
	Volta	123	6.3%	95	9.7%	28	2.9%	59	62.2%	36	37.8%	19	67.7%	9	32.3%
	Western	207	10.7%	145	14.9%	62	6.4%	103	71.1%	42	28.9%	31	49.1%	32	50.9%
Facility type visited															
	CHPS	210	11.0%	202	21.0%	8	0.9%	189	93.6%	13	6.4%	7	86.3%	1	13.7%
	Govt. Health center	464	24.2%	317	33.1%	146	15.4%	247	77.9%	70	22.1%	119	81.4%	27	18.6%
	Govt. Hospital/polyclinic	777	40.6%	292	30.4%	485	50.9%	140	47.9%	152	52.1%	311	64.2%	174	35.8%
	Other	108	5.7%	65	6.8%	43	4.5%	48	74.0%	17	26.0%	30	68.5%	14	31.5%
	Private hospital/clinic	353	18.5%	83	8.6%	271	28.4%	34	41.0%	49	59.0%	168	62.0%	103	38.0%
Owns a bicycle															
	no	1445	74.2%	674	69.2%	770	79.3%	453	67.2%	221	32.8%	506	65.7%	264	34.3%
	yes	501	25.8%	301	30.8%	201	20.7%	216	72.0%	84	28.0%	142	70.5%	59	29.5%
Owns car															
	no	1764	90.7%	942	96.6%	823	84.7%	652	69.2%	290	30.8%	550	66.8%	273	33.2%
	yes	182	9.3%	33	3.4%	148	15.3%	18	53.1%	16	46.9%	98	65.8%	51	34.2%
Paid fees for family planning in the last 12 months															
	no	298	41.1%	148	37.8%	150	44.8%	104	69.8%	45	30.2%	84	56.2%	66	43.8%
	yes	428	58.9%	243	62.1%	185	55.2%	182	74.9%	61	25.1%	134	72.6%	51	27.4%
Confidence that if you became very sick tomorrow, that you would be able to receive effective treatment from the health system															

© 2020 Bell G et al. *JAMA Network Open*

	not at all confident	56	2.9%	33	3.4%	23	2.4%	29	87.5%	4	12.5%	13	56.9%	10	43.1%
	not very confident	135	7.0%	92	9.5%	43	4.4%	67	72.2%	26	27.8%	30	69.8%	13	30.2%
	somewhat confident	372	19.2%	178	18.3%	194	20.0%	133	74.5%	45	25.5%	128	65.8%	66	34.2%
	very confident	1378	71.0%	669	68.8%	710	73.1%	438	65.6%	230	34.4%	477	67.2%	233	32.8%
Taken care of by the same provider each time															
	always	438	22.6%	258	26.6%	180	18.6%	203	78.4%	56	21.6%	129	71.9%	51	28.1%
	frequently	575	29.6%	324	33.3%	251	25.9%	217	67.0%	107	33.0%	165	65.8%	86	34.2%
	rarely	766	39.4%	297	30.5%	469	48.4%	194	65.3%	103	34.7%	314	66.8%	156	33.2%
	never	163	8.4%	93	9.6%	70	7.2%	54	58.0%	39	42.0%	38	54.6%	32	45.4%
Easy or difficult to pay for visit															
	very difficult	200	10.3%	145	14.9%	55	5.7%	86	59.6%	58	40.4%	29	52.0%	26	48.0%
	difficult	373	19.3%	203	20.8%	171	17.7%	127	62.9%	75	37.1%	103	60.4%	68	39.6%
	easy	863	44.5%	382	39.3%	481	49.8%	283	73.9%	100	26.1%	345	71.9%	135	28.1%
	very easy	500	25.8%	243	25.0%	257	26.7%	171	70.4%	72	29.6%	164	63.5%	94	36.5%
Have health insurance															
	no	485	24.9%	249	25.6%	235	24.2%	168	67.3%	82	32.7%	156	66.1%	80	33.9%
	yes	1462	75.1%	726	74.4%	736	75.8%	502	69.2%	224	30.8%	492	66.9%	244	33.1%
Had to borrow money to pay for visit															
	no	1558	80.2%	711	72.9%	847	87.5%	508	71.5%	203	28.5%	572	67.6%	275	32.4%
	yes	385	19.8%	264	27.1%	122	12.6%	161	61.1%	103	38.9%	73	60.2%	48	39.8%
Rating of the amount of time provider spent with you															
	excellent	320	16.4%	182	18.7%	138	14.2%	105	57.6%	77	42.4%	85	62.0%	52	38.0%
	very good	608	31.2%	277	28.5%	330	34.0%	182	65.7%	95	34.3%	215	65.0%	116	35.0%
	good	857	44.1%	430	44.1%	427	44.0%	322	75.0%	108	25.0%	297	69.4%	131	30.6%
	fair	138	7.1%	77	7.8%	62	6.3%	52	68.4%	24	31.6%	41	66.6%	21	33.4%
	poor	23	1.2%	9	0.9%	14	1.5%	8	86.3%	1	13.7%	10	69.2%	4	30.8%
Sought care for self															
	no	599	30.8%	305	31.3%	294	30.3%	230	75.4%	75	24.6%	205	69.8%	89	30.2%
	yes	1348	69.2%	670	68.7%	677	69.8%	440	65.6%	230	34.4%	443	65.3%	235	34.7%
Sought care for child															

© 2020 Bell G et al. *JAMA Network Open*

	no	1143	58.7%	535	54.8%	608	62.6%	344	64.3%	191	35.7%	401	65.9%	207	34.1%
	yes	804	41.3%	440	45.2%	363	37.4%	326	74.0%	114	26.0%	247	67.9%	117	32.1%
Sought care for other															
	no	1841	94.6%	932	95.6%	909	93.6%	639	68.6%	293	31.4%	606	66.7%	303	33.3%
	yes	106	5.4%	43	4.4%	63	6.5%	30	70.2%	13	29.8%	42	66.7%	21	33.3%
Amount paid out of pocket for visit, mean (SE)															
		73.8	14.2	42.5	5.4	105.5	27.8	26.8	3.8	76.9	14.6	80.6	24	154.8	37.5%

© 2020 Bell G et al. *JAMA Network Open*

eTable 2. Reasons Given for Why Women Bypassed Their Nearest Facility

	No.	%
Did not visit closest facility due to no services		
no	245	51.1
yes	234	48.9
Total	479	100
Did not visit closest facility due to facility being closed		
no	442	92.2
yes	37	7.8
Total	479	100
Did not visit closest facility because they had already gone to that facility		
no	381	79.6
yes	98	20.4
Total	479	100
Did not visit closest facility due to high cost		
no	438	91.4
yes	41	8.6
Total	479	100
Did not visit closest facility due to reachability		
no	441	91.9
yes	39	8.1
Total	479	100
Did not visit closest facility due to distrust		
no	442	92.2
yes	38	7.8
Total	479	100
Did not visit closest facility due to a previous bad experience		
no	435	90.8
yes	44	9.2
Total	479	100
Did not visit closest facility due to privacy		
no	446	93

© 2020 Bell G et al. *JAMA Network Open*

yes	34	7
Total	479	100

© 2020 Bell G et al. *JAMA Network Open*
